# Developing a Web-Based Shared Decision-Making Tool for Fertility Preservation Among Reproductive-Age Women With Breast Cancer: An Action Research Approach

**DOI:** 10.2196/24926

**Published:** 2021-03-17

**Authors:** Ling-Ming Tseng, Pei-Ju Lien, Chen-Yu Huang, Yi-Fang Tsai, Ta-Chung Chao, Sheng-Miauh Huang

**Affiliations:** 1 Comprehensive Breast Health Center Department of Surgery, Taipei Veterans General Hospital Taipei Taiwan; 2 School of Medicine National Yang Ming Chiao Tung University Taipei Taiwan; 3 Division of Reproductive Endocrinology and Infertility Department of Obstetrics and Gynecology Taipei Veterans General Hospital Taipei Taiwan; 4 Division of Medical Oncology Department of Oncology Taipei Veterans General Hospital Taipei Taiwan; 5 MacKay Medical College Department of Nursing New Taipei City Taiwan

**Keywords:** breast cancer, shared decision making, website, action research, fertility preservation

## Abstract

**Background:**

The pregnancy rate after cancer treatment for female survivors is lower than that of the general population. Future infertility is a significant concern for patients with breast cancer and is associated with a poor quality of life. Reproductive-age patients with breast cancer have safe options when choosing a type of fertility preservation method to be applied. Better information and support resources aimed at women to support their decision making are needed.

**Objective:**

The objective of this study was to develop a web-based shared decision-making tool for helping patients with breast cancer make decisions on fertility preservation.

**Methods:**

We used the action research cycle of observing, reflecting, planning, and acting to develop a web-based shared decision-making tool. The following four phrases were applied: (1) observe and reflect—collect and analyze the decision-making experiences of patients and health care providers; (2) reflect and plan—apply the initial results to create a paper design and modify the content; (3) plan and act—brainstorm about the web pages and modify the content; (4) act and observe—evaluate the effectiveness and refine the website’s shared decision-making tool. Interviews, group meetings, and constant dialogue were conducted between the various participants at each step. Effectiveness was evaluated using the Preparation for Decision-Making scale.

**Results:**

Five major parts were developed with the use of the action research approach. The Introduction (part 1) describes the severity of cancer treatment and infertility. Options (part 2) provides the knowledge of fertility preservation. The shared decision-making tool was designed as a step-by-step process (part 3) that involves the comparison of options, patient values, and preferences; their knowledge regarding infertility and options; and reaching a collective decision. Resources (part 4) provides information on the hospitals that provide such services, and References (part 5) lists all the literature cited in the website. The results show the web-based shared decision-making meets both patients’ and health providers’ needs and helps reproductive-age patients with breast cancer make decisions about fertility preservation.

**Conclusions:**

We have created the first web-based shared decision-making tool for making fertility preservation decisions in Taiwan. We believe female patients of reproductive age will find the tool useful and its use will become widespread, which should increase patient autonomy and improve communication about fertility preservation with clinicians.

**Trial Registration:**

Clinicaltrials.gov NCT04602910; https://clinicaltrials.gov/ct2/show/NCT04602910

## Introduction

Breast cancer is a common oncologic disease worldwide. In Taiwan, breast cancer has the highest incidence of all cancers in the female population, and the incidence of breast cancer in women of childbearing age is increasing [1]. Approximately 12% to 19% of patients are affected when they are of reproductive age [1-3]. Fertility and infertility are important concerns among reproductive-age women with breast cancer [4,5]. Infertility following cancer treatment has a recognized negative impact on the quality of life [6]. The National Cancer Institute (Taiwan) defined fertility preservation as a type of procedure used to help a person’ retain the ability to have children [7]. The key point is that reproductive-age patients have more than one fertility preservation choice. Unfortunately, most patients do not have enough information to make an informed decision prior to cancer treatment. For instance, ovarian stimulation in patients with early-stage breast cancer is safe in the long term [3]. Women diagnosed with cancer who have eggs or embryos cryopreserved before anticancer treatment have good chances for successful assisted reproductive technology performance and good perinatal outcomes [8-10]. In addition, the efficacy and safety of temporary ovarian suppression with gonadotropin-releasing hormone agonist during chemotherapy might be an available option to reduce the likelihood of chemotherapy-induced primary ovarian insufficiency and to improve future fertility in premenopausal patients with early breast cancer [11]. It is worth noting that there is consensus in medical society that the long-term survival rate of the disease is not lower for any of the three types of fertility preservation [8-11]. Thus, if age, reproductive function, and the cancer stage allow, patients can choose their preferred fertility preservation method.

A previous study [12] indicated patients’ decisions about fertility are multidimensional. The risk perception of pregnancy among patients with breast cancer after treatment focused on “reaching the balance of life [12].” Women treated for breast cancer applied risk-benefit perceptions to decide whether to become pregnant [12]. There are several factors related to the ability to make well-informed decisions regarding fertility preservation. First, patients with a new diagnosis of breast cancer often experience negative emotions, such as anxiety, depression, and uncertainty, due to their perceptions of life-threatening cancer [13-15]. Second, women are expected to have biological children because of the Chinese cultural belief in lineal descent from one’s ancestors, which is deeply rooted among the Taiwanese people. The assignment of great importance to parenthood was directly associated with higher depression symptoms in reproductive-age women with breast cancer [16].

Additionally, some health care providers might keep silent about discussing fertility because their primary concern is getting their patients to be cancer free [17]. Patients with breast cancer lacked easily available knowledge about infertility and underestimated the possibility of infertility [18]. This requirement for information is mostly unmet. Patients do not fully understand the impact of cancer treatment and the possibility of infertility because of treatment. Finally, the space and environment of the outpatient clinic are not conducive to the discussion of private issues. Fertility concerns are not encouraged to be brought to the physicians’ or nurses’ attention before cancer treatment. Although women want to request further information regarding future fertility, they may hesitate even when talking to their physicians, disclosing their opinion, or showing their personal feelings.

The first Patient Autonomy Act was passed through the Legislative Yuan in Taiwan in 2015. Now, it is vital to ensure that patients have the right to know, choose, or refuse medical care, and in addition to being informed, patients can choose their medical options. All of the above can be achieved via shared decision making (SDM) [19-22]. The Joint Commission of Taiwan, which is an organization established in 1999 with funding from the Ministry of Health and Welfare, Taiwan Hospital Association, promotes the development of SDM [23]. Difficulty accessing decision aids or limited patient comprehension were common barriers to realizing shared decision making [24]. Decision aids that improve decision-related outcomes for many breast cancer treatment decisions, including surgery, radiotherapy, and endocrine and chemotherapy, are available [25]. Fertility preservation with decision aids is well-developed in some economically high-income countries, such as Australia [26], Canada [27], Netherlands [28], and the United States [29]. Therefore, we aimed to develop a web-based SDM tool for fertility preservation for patients with breast cancer. The web-based decision aid was expected to provide medical information, help the patient explore and compare treatment options, assess the patient’s values and preferences, and reach a collective decision on fertility preservation.

## Methods

### Research Design

We applied an action research design, which is defined as an approach that involves collaboration to develop a process through knowledge-building and social change [30]. Learning by doing with the participants, including the patients, is the heart of health care action research. The action research approach is particularly relevant when treating patients with chronic diseases and complex care needs [31,32]. Previous investigators have applied action research to build web-based comics [33] or decision-aid websites [34] about breast cancer surgery in Taiwan. However, few study the decision-making and research processes for oncofertility in Asia. Therefore, the ultimate goal of this study was to find supporting evidence of the benefits to patients and families of a web-based platform that assists patients with breast cancer in the SDM for fertility preservation.

### Project Team

The SDM team was multidisciplinary, comprising a senior researcher, two surgeons, an oncologist, a gynecologist, an advanced nurse specialist, and an information technology (IT) engineer. The surgeon, oncologist, and gynecologist were responsible for gathering and reviewing existing fertility preservation literature to create easy-to-understand formats and simple graphic renderings of patients with breast cancer. The researcher and nurse specialist interviewed women and health providers to explore their experiences regarding the decision-making process. The IT engineer was responsible for informatics work related to website page design, program coding, and hosting of the website. The members involved in breast care had at least 10 years of experience. The IT engineer was a senior website designer with 5 years of work experience. The team had regular face-to-face and line meetings to prepare for and reflect on the development of the oncofertility SDM program and the community of fertility preservation practice. The SDM team was the partnership in the sense of doing together and deciding together during the research phase. The roles of the members were alternately those of initiator, educator, facilitator, coach, and finally, coauthors.

### Action Research Process

**Figure 1:**
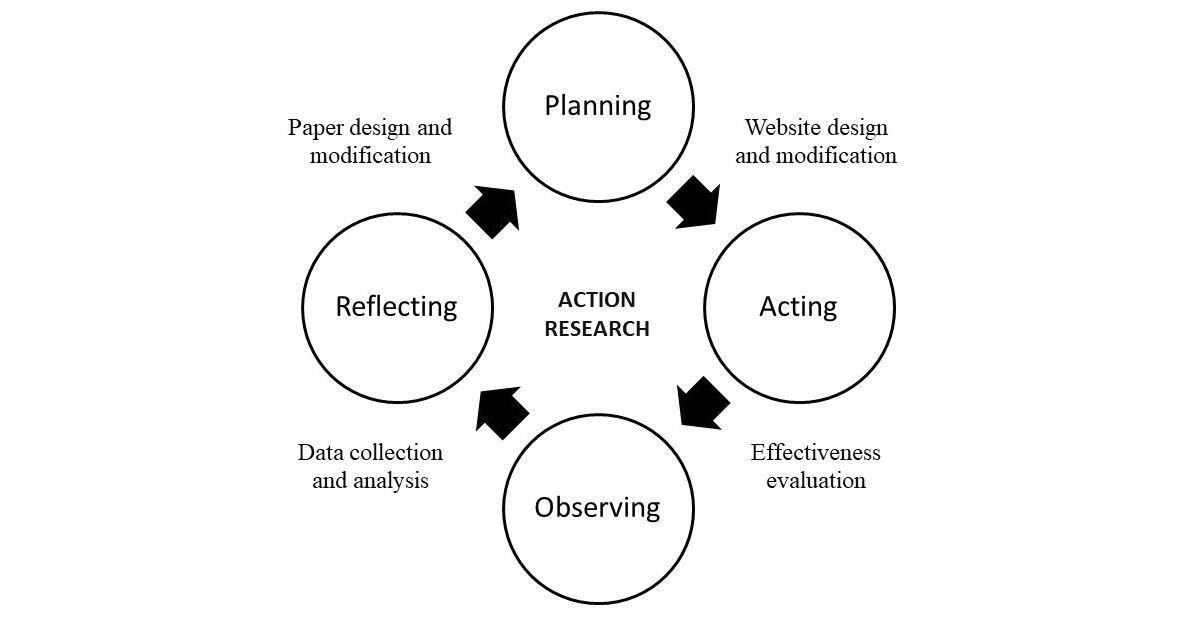
The process of action research in developing web-based shared decision-making regarding fertility preservation.

A participatory action research methodology was used to facilitate the development of the SDM program for the multidisciplinary care team at the same time that a web-based SDM of fertility preservation practice was being established. The action research cycle of observing, reflecting, planning, and acting was applied in this study (Figure 1).

Because the SDM involved both health care providers and patients, we collected data from both views at the phases of observing and reflecting. Health care providers were recruited to interview and ask patients about their experiences regarding fertility. We also recruited breast cancer survivors to explore the decision-making experience of pregnancy. The reflections were viewed as a continuous dialogue among team members during the SDM program. The results of data collection and team self-evaluation of our positionality were analyzed, with the explicit recognition that this position may affect the SDM process and outcome. The evaluations were interdependent and partly based on the project team members’ interpersonal, social, and institutional contexts.

During the phases of reflecting and planning, all major team members not only reviewed the health issues and evidence-based literature related to fertility preservation in patients with cancer but also discussed the SDM content based on the findings from the observe and reflect phases. Three patients were invited to participate and provide their suggestions. The initial paper-designed content of SDM for fertility preservation was formed by consensus after 6 meetings.

During the phases of planning and acting, the initial website structure was developed according to the 5 steps of SDM. Each part of the SDM was assessed for comprehensibility and usability by patients. Health care providers were asked to assess the acceptability of the SDM content. A 5-point Likert scale was used for patient and health care provider ratings for each item. If the score was less than 3, then we modified the website content based on the user feedback from patients and health care providers.

During the phase of acting, we evaluated the effectiveness of the Preparation for Decision-Making (prepDM) scale developed by Bennett et al [35]. The Joint Commission of Taiwan also suggested the effects of SDM be evaluated in patients and health care providers. Both patients and health care providers were invited to answer 10 questions by responding with a 5-point Likert scale rating (from 1 to 5). A higher score indicated greater agree with the effect.

### Setting

The setting was the comprehensive breast health center at Taipei Veterans General Hospital in Taipei, Taiwan. The center integrates multidisciplinary care to provide comprehensive services, including diagnosis and treatment, to achieve the purpose of patient-centric care. The health care providers included surgeons, gynecologists, Chinese medicine doctors, nurses, psychological consultants, social workers, and nutritionists. The web-based SDM for fertility preservation was linked to the hospital website.

### Analysis

In-depth interviews were done to collect and gain deep insights into the views and needs of the participants. Data collected through the face-to-face interviews were transcribed from audiorecordings to written transcripts. The content analysis method was used to analyze the data. We checked whether the categories were stable and provided sufficient depth, then multiple strategies were applied to ensure trustworthiness. The discussion and decision at the group meetings were also recorded. The effectiveness was evaluated using the prepDM scale developed by Bennett et al [35]. Statistical analyses were performed using the Predictive Analytics Suite workstation (version 18.0; IBM Corp). Individual variables were examined by percentages, means, and standard deviations.

## Results

### Results of the Observe and Reflect Phase

Cancer survivors (n=16) were recruited to explore the decision-making experience of determining whether to have a pregnancy (from 2015 through 2016). The result showed decision making regarding fertility among women with cancer was affected by preexisting needs for children before treatment and their experience during treatment. Underestimating the possibility of infertility and a lack of knowledge of fertility preservation might cause the woman to regret her decision. (Participant names are pseudonyms.)

If I had known in advance, I could have frozen my eggs. I mean, normal eggs are taken before chemotherapy. Because it [chemotherapy] has damaged my eggs now, I can’t afford another invasive examination or treatment.

Bella

Health care providers (n=16), including nurses (n=8), surgeons (n=3), gynecologists (n=2), Chinese medicine doctor (n=1), psychology consultant (n=1), and social worker (n=1), were also recruited to interview and ask the women about their experiences regarding fertility (from 2017 through 2018). Although most of the health care providers were concerned about the severity of the cancer and the urgency of treatment, they agreed to satisfy the women’s needs and respect their choice. However, there are some barriers to be solved, such as communication blocks among the multidisciplinary team, lack of initial screening, and lack of evidence-based information regarding oncofertility.

I think the empirical literature may exist, but nobody has assembled it into a simple language that the patient can understand. All we know is that it requires more manpower and material resources...

Jeff

### Results of the Reflect and Plan Phase

**Figure 2:**
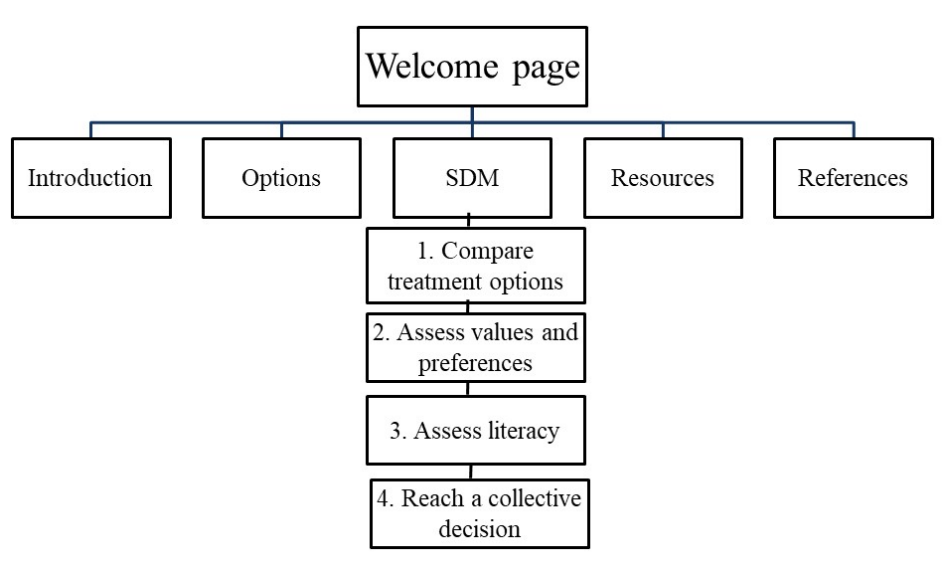
Website structure. SDM: shared decision making.

After collecting the data and performing a content a qualitative content analysis, we discussed strategies to overcome the barriers. The SDM process was expected to progress step by step, according to the five essential steps of SDM developed by the Agency for Healthcare Research and Quality [36]. Reproductive-age women had insufficient knowledge regarding infertility and the types of fertility preservation. More than 96% of Taiwanese ever used the internet to search for information [37]. Hence, all project members focused the information they provided on the website on cancer treatment, infertility, and the types of fertility preservation necessary before embarking on the SDM steps. Therefore, the introduction involved the description of the severity of the cancer and its treatment and the possibility of infertility. Then, the options regarding fertility preservation were designed. The SDM part included providing comparisons of treatment and fertility preservation options, assessing each patient’s values and preferences (such as cost or safety), assessing their degree of knowledge regarding infertility and fertility preservation options, and reaching a collective decision on the best option. We also linked the organizations that provide fertility preservation services in a resource section [38]. Appropriate literature was also provided in the reference section. The website structure was designed, as shown in Figure 2. All team members were assigned to fulfill this information need for the patients. The content provided in each section was reached by consensus in group meetings.

### Results of the Plan and Act Phase

Upon entering the welcome page, patients are shown a clear title; for example, “I am a patient with breast cancer. How will I fulfill my dream of having a child? (Chinese: 我是乳癌病人，怎麼圓生子夢?).” Our webpage follows a structure where the five main parts with their corresponding submenus are displayed as the user clicks on them (Multimedia Appendix 1). The five main webpage subtitles are Introduction, Options, SDM, Resources, and Reference Links. A 5-point Likert scale was used for patient and care provider ratings. Patients (n=13) rated the web-based SDM for comprehensibility (mean 3.9, SD 0.9) and usability (mean 3.9, SD 1.1). Health care providers (n=9) were also asked to assess the acceptability (mean 4.3, SD 0.8). If the score was less than 3, we modified the website content based on the user feedback from patients and health care providers. For example, patients mentioned that pharmaceutical and scientific names or brand names (such as gonadotropin-releasing hormone agonist or Zoladex) are too hard to understand. Patients said it is easier to understand simpler terms like “medicine postponing menstruation” (Chinese: 注射停經針). When patients were tested on their knowledge regarding fertility preservation, some still misunderstood and felt frustrated. Hence, we provide links to help answer questions and clarify their knowledge. When all questions are answered correctly, the user can enter the next stages (Multimedia Appendix 2; Multimedia Appendix 3; Multimedia Appendix 4).

The Action Script technology for the front-end framework was Vue.js, and the back-end framework was Laravel 5 (requiring PHP 7.2). The operating system was Amazon Linux 2, while the network server was Apache 2.4. AWS EC2 hosted the web-based machine. The database was MySQL 5.7 (Amazon RDS).

### Results of the Act and Observe Phase

We used a multifaceted approach to evaluate the website after the completion of the preliminary website. The SDM effectiveness of the website was evaluated using the SDM questionnaire developed by The Joint Commission of Taiwan. The participants in the evaluation consisted of 14 reproductive-age patients with breast cancer (n=14, prepDM: mean 4.1, SD 0.8) and 11 hospital staff (n=11, prepDM: mean 4.2, SD 0.7) (Table 1). Both patients and staff had the perception that the web-based SDM was effective for helping reproductive-age patients with breast cancer make fertility preservation decisions. Based on feedback from the patients and hospital care providers, we refined the options because some patients had hesitation about the website. The option “can’t decide right now” was added, and qualitative and quantitative items regarding further needs were added to the survey (Multimedia Appendix 5).

**Table 1:** Results of the Preparation for Decision-Making Survey scale among health care providers and patients with breast cancer.

Item	Health providers (n=11), mean (SD)	Patients (n=14), mean (SD)
1. Recognize decision needs to be made	4.1 (0.8)	4.1 (0.9)
2. Prepare my patient/you to make a better decision	4.2 (0.9)	4.2 (1.0)
3. Think about pros and cons of each option	4.2 (0.8)	4.2 (0.8)
4. Help my patient/you think about which pros and cons are most important	4.3 (0.6)	4.3 (0.8)
5. Know that decision depends on what matters most to my patient/you	4.1 (0.8)	4.2 (0.8)
6. Organize my patient’s/your thoughts about decision	4.2 (0.8)	3.9 (1.0)
7. Think about how involved my patient/you want to be in the decision	4.4 (0.8)	3.9 (0.9)
8. Help my patient/you identify questions you want to ask	4.4 (0.7)	4.1 (0.9)
9. Talk to patient’s/your doctor about what matters most	4.3 (1.0)	4.1 (1.0)
10. Promote health literacy/prepare you for a follow-up visit with your doctor	4.3 (0.8)	3.9 (1.2)
Average	4.2 (0.7)	4.1 (0.8)

## Discussion

### Principal Findings

We used action research to create a website for the SDM model for fertility preservation among young women with breast cancer [39]. The approach consisted of four phases based on the principles of SDM. Previous SDM-related literature has highlighted the importance of the principles of informed consent and patient autonomy [19-22]. When women make a meaningful decision regarding their choice of fertility preservation, their degree of medical literacy, preferences, and a close relationship (mutual communication and understanding) between themselves and health care providers are often key challenges. Sometimes, a conflict happens between what the patients want and what the clinicians deem necessary in terms of the website during the four phases. We not only collected qualitative and quantitative data but brainstormed acceptable content for both health care providers and patients. This work implied that the SDM for fertility preservation, as guided by action research, would bridge the gap between the patients and clinicians and caters more toward the feelings and needs of the patients’ minds and bodies than previous approaches. The findings were consistent with those of previous studies [33,34] for which decision-aid websites for breast cancer surgery or comics for newly diagnosed breast cancer in women were developed. Compared with the development of medical information websites, the development of web-based decision aids using action research may be more systematic and humanistic because they account for both the patients’ and health care providers’ perspectives and needs. When considering the participants’ corresponding culture and the region in which they live, our study serves as a reference for describing how to develop decision support tools for women with breast cancer and fertility needs.

Limited health literacy is associated with poorer health outcomes and may make it difficult for cancer patients to participate in the SDM process [23]. Increasing health literacy has the potential to increase health care engagement and, subsequently, to increase the use of SDM [40]. Improving women’s health literacy is a necessary strategy to promote informed consent and SDM. In Taiwan, explaining the disease condition and its related treatment is the responsibility of the attending physician. Physicians explain the disease and related treatments in colloquial language, which is a key factor in promoting patients’ perceived involvement in SDM [41]. Before the SDM process, our Introduction and Options website pages provided familiar text and pictures for physicians to explain the consequences of infertility and the methods of fertility preservation. It was also easy for patients to go back and reread the pages for better understanding. Previous scholars mentioned three major SDM steps, which included exploring care or treatment options and their risks and benefits, discussing choices available, and reaching a decision about care or treatment, together with their health and social care professionals [21,42]. In our study, we added the literacy assessment before reaching a collective decision. The assessment enabled us to make sure our participants had enough knowledge regarding infertility and fertility preservation before making a decision. The impact of the health literacy intervention on decision regret and psychological changes merits future research.

### Limitations

There were several challenges during the development of the website. The patient’s psychological burden and fear increased with the severity of the cancer stage. Despite this situation, we did not provide different options for fertility preservation for patients with severer cancer stages because of insufficient evidenced-based literature. Evidence-related survival rates and recurrence rates among women with fertility preservation require more research.

With advancements in reproductive technologies, the evidence will be updated, and new options may appear periodically when the website is updated. The action research cycle provides an opportunity to modify and confirm this repeatedly, as it is quite a repetitive and lengthy process for the professionals involved.

The SDM webpages were limited to the Chinese version. Hence, we could not explore the advantages and disadvantages related to non-Chinese and non-English literature and websites. The web-based SDM regarding fertility preservation was only designed for women with breast cancer. Nonetheless, this study can serve as a foundation and reference for health care providers who are interested in future planning, promotion, operation, and long-term management of websites for patients with cancers other than breast cancer. The SDM website was hosted by the hospital and depended on internal technical support. All researchers in our study are responsible for ensuring a responsive-interactive website. Considering information security, patients need to provide some personal information to enter the welcome page. If the patient uses the website alone and replies to the questions on the webpage, a time delay in getting a doctor’s response may increase the patient’s anxiety because patients and clinicians are encouraged to visit the website together so that the doctor can answer any questions. We only evaluated the effectiveness of web-based SDM using the prepDM scale. Using the full-fledged website evaluation heuristics and exploring the long-term consequences, such as decision regret and quality of life among reproductive-age women with breast cancer, merit more study in the future.

### Conclusions

With the help of the study results, we built a web-based decision-aid tool for helping reproductive-age women with breast cancer make decisions on fertility preservation. The action research provided a good structure to guide cooperation involving patients, clinicians, nurses, academic researchers, and IT engineers. The research results helped our team manage effectively and shortened the distance between theory and practice. It also helped participants who are facing the dilemma of choosing a method of fertility preservation. Based on the importance of informed consent, our website not only provided knowledge of infertility and the methods of fertility preservation but allowed us to design and test this information. It helps ensure that our participants make fertility preservation decisions with knowledge and understanding. Based on our findings, we conclude that this SDM website can indeed help patients with early-stage breast cancer make more informed decisions regarding the type of fertility preservation they would prefer to undergo. Longitudinal studies to follow up on the changes in psychological condition and subsequent pregnancy rates are needed in the future.

## Appendix

Multimedia Appendix 1 Structure of 5 main parts in the first shared decision-making webpage.

Multimedia Appendix 2 Step 1 of the SDM process.

Multimedia Appendix 3 Step 2 of the SDM process.

Multimedia Appendix 4 Step 3 of the SDM process.

Multimedia Appendix 5 Step 4 of the SDM process.

## Abbreviations

IT: information technology
PrepDM: Preparation for Decision-Making scale
SDM: shared decision making
