# Supplementary figures and images for: Developing a Web-Based Shared Decision-Making Tool for Fertility Preservation Among Reproductive-Age Women With Breast Cancer: An Action Research Approach
Source: J Med Internet Res. 2021 Mar 17;23(3):e24926. doi: 10.2196/24926 (PMC8074988; doi:10.2196/24926)

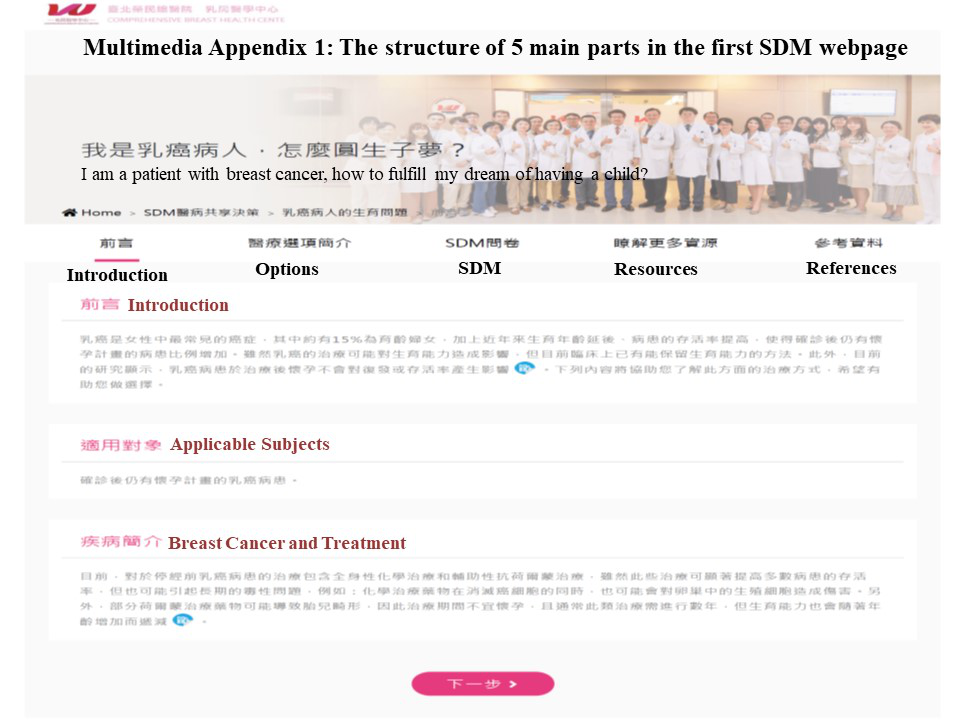

Supplement: Multimedia Appendix 1 [file jmir_v23i3e24926_app1.png]

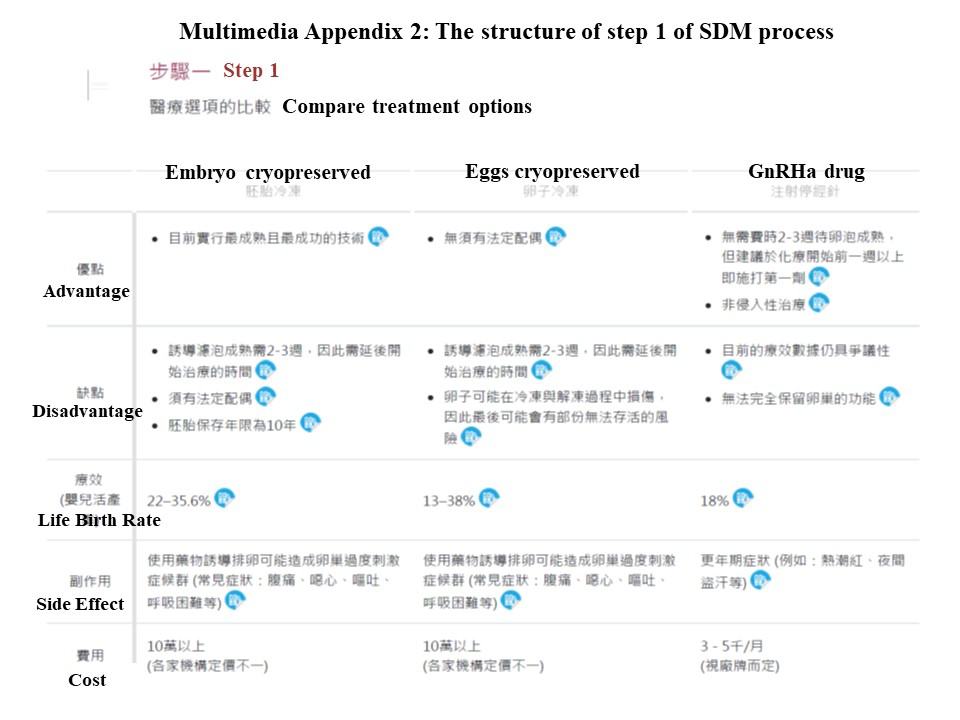

Supplement: Multimedia Appendix 2 [file jmir_v23i3e24926_app2.png]

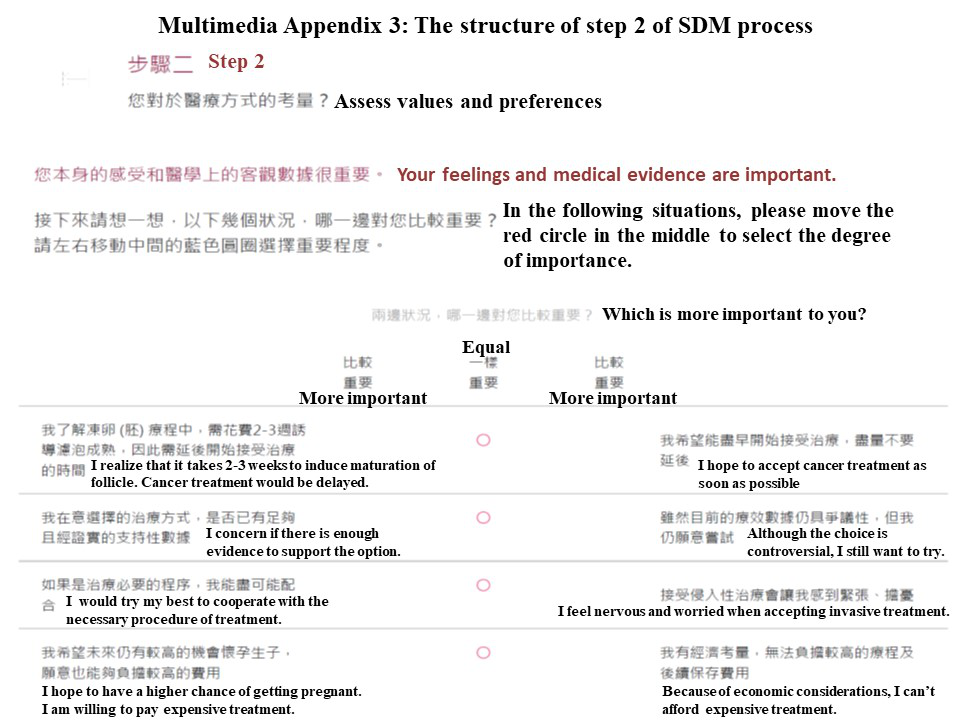

Supplement: Multimedia Appendix 3 [file jmir_v23i3e24926_app3.png]

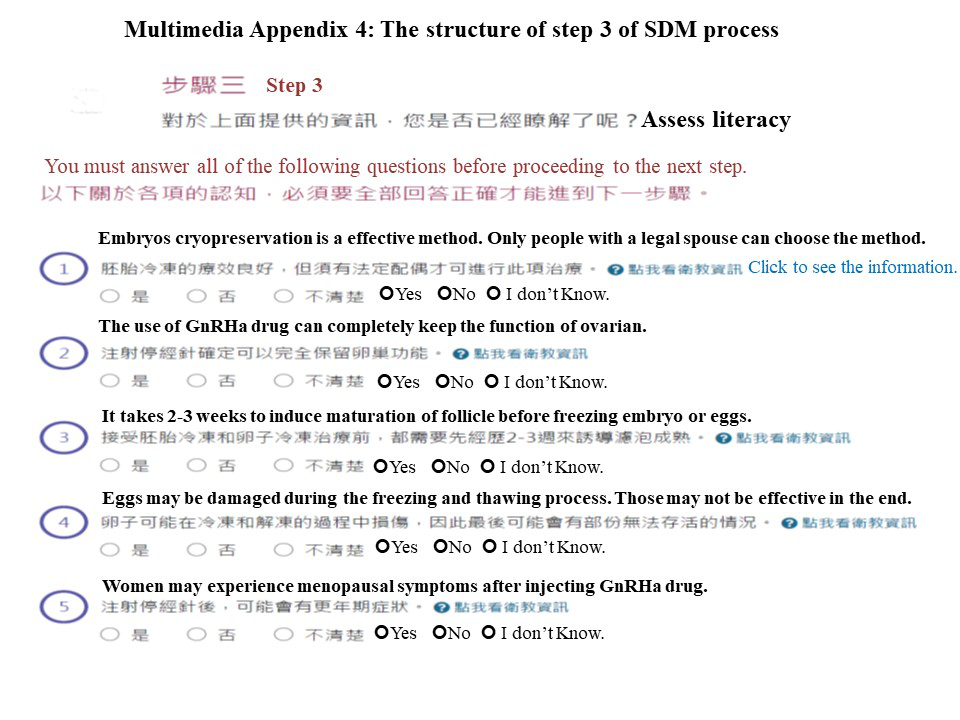

Supplement: Multimedia Appendix 4 [file jmir_v23i3e24926_app4.png]

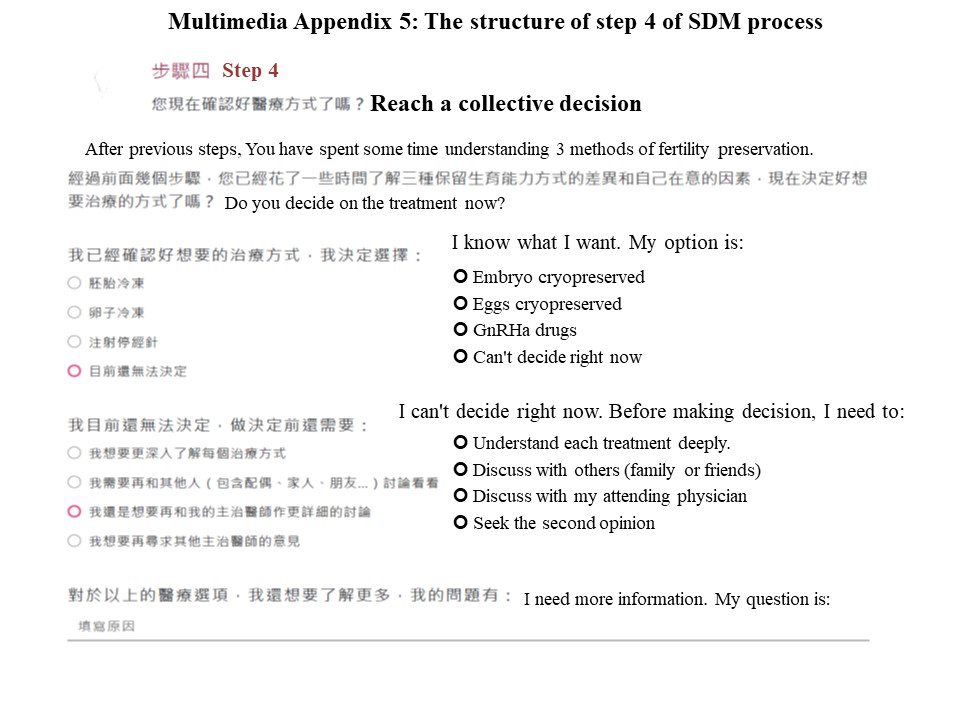

Supplement: Multimedia Appendix 5 [file jmir_v23i3e24926_app5.png]
